# Relative entropy differences in bacterial chromosomes, plasmids, phages and genomic islands

**DOI:** 10.1186/1471-2164-13-66

**Published:** 2012-02-10

**Authors:** Jon Bohlin, Mark WJ van Passel, Lars Snipen, Anja B Kristoffersen, David Ussery, Simon P Hardy

**Affiliations:** 1 Norwegian School of Veterinary Science, EpiCentre, Department of Food Safety and Infection biology, Ullevålsveien 72, Oslo, Norway; 2 Systems and Synthetic Biology, Wageningen University, Wageningen, the Netherlands; 3 Norwegian University of Life Sciences, Department of Chemistry, Biotechnology and Food Sciences, Ås, Norway; 4 National Veterinary Institute, Ullevålsveien 68, Pb 750 Sentrum, N-0106 Oslo, Norway; 5 Center for Biological Sequence Analysis, Department of Systems Biology, Comparative genomics unit, Technical University of Denmark, DK-2800 Lyngby, Denmark

## Abstract

**Background:**

We sought to assess whether the concept of relative entropy (information capacity), could aid our understanding of the process of horizontal gene transfer in microbes. We analyzed the differences in information capacity between prokaryotic chromosomes, genomic islands (GI), phages, and plasmids. Relative entropy was estimated using the Kullback-Leibler measure.

**Results:**

Relative entropy was highest in bacterial chromosomes and had the sequence chromosomes > GI > phage > plasmid. There was an association between relative entropy and AT content in chromosomes, phages, plasmids and GIs with the strongest association being in phages. Relative entropy was also found to be lower in the obligate intracellular *Mycobacterium leprae *than in the related *M. tuberculosis *when measured on a shared set of highly conserved genes.

**Conclusions:**

We argue that relative entropy differences reflect how plasmids, phages and GIs interact with microbial host chromosomes and that all these biological entities are, or have been, subjected to different selective pressures. The rate at which amelioration of horizontally acquired DNA occurs within the chromosome is likely to account for the small differences between chromosomes and stably incorporated GIs compared to the transient or independent replicons such as phages and plasmids.

## Background

Horizontal gene transfer in microbial communities has been recognized as a key driver of evolutionary change in microbes [1,2]. In addition to plasmids and phages, regions within the bacterial chromosomes are assumed to have been horizontally acquired [3]. Such putatively horizontally transferred regions are termed Genomic Islands (GI). GIs originate from different sources [4] including plasmids and phages (prophages) and carry traits that have important biological phenotypes such as virulence determinants and antibiotic resistance genes. Genetic material is most readily exchanged between related genetic elements, [5]*i.e*. chromosomes exchange DNA with chromosomes, plasmids with plasmids, and phages with phages. However, this exchange is not entirely restrictive with low frequency transfer occurring between chromosomes on one hand and plasmids and phages on the other [5]. Mathematical models predict plasmids to be the predominant means of genetic variation among bacteria [5]. Based on findings from genomic signatures (and analyses of CRISPSs in bacteria [6]), phages, and viruses in general, have been found to co-evolve with their hosts [7]. Plasmids on the other hand, although sharing some similarities with their hosts, have a more different DNA composition than what would be expected compared to the hosts chromosome [8]. In fact, genomic signatures based methods reveal prokaryotic plasmid-host similarity to correlate with genomic GC content, *i.e*. the more GC rich an organism is the more compositionally similar it tends to be with its plasmid(s) [9]. GC content has also been associated with genome wide rates of mutation, where organisms of low GC content tend to have more random genomes than GC rich ones [10,11], *i.e*. the signal-to-noise ratio is lower in AT rich genomes. An organism's DNA sequence that has been subjected to numerous random mutations is assumed to possess less information than the DNA of an organism under strong selective pressure. In other words, due to more accumulated mutations, it appears as if less information is carried by the DNA sequences of AT rich microbes compared to GC rich microbes. Thus, to test the assertion that accumulated mutations lower the information capacity we explored the use of information theory as a means of measuring information capacity in DNA sequences.

The concept of information theory was originally introduced by Claude E. Shannon as a tool to systematically analyze data flow in general communication systems [12]. The theory has been extended and subsequently applied to many fields including DNA sequence analysis [13-15]. Methods of Information theory focusing on DNA sequence compression have found differences between coding and non-coding sequences as well as between prokaryotic and eukaryotic organisms [16].

These results led us to apply information theoretical methods to examine the extent to which information content differed between the genomes of bacterial chromosomes, plasmids, phages and GIs, and whether such differences could be related to distinct genomic properties of bacterial chromosomes and mobile genomic elements. We used the Kullback-Leibler divergence measure (*D_KL_*) of tetranucleotide frequencies within genomic DNA sequences, similar to that descried by Sadovsky [15], but using tetranucleotide frequencies and a zero order Markov model instead of a second order Markov model. These alterations increase the sensitivity of detection [17]. The zero order Markov model assumes the simplest possible dependence structure between neighboring nucleotides. This means that *D_KL _*will be higher than in models that do account for dependence between adjacent nucleotides, like the first or second order Markov models [17]. The expected tetranucleotide frequencies, statistically speaking, are thus calculated from mononucleotide frequencies implying that the bases are independent of each other. Thus, *D_KL _*reflects relative entropy in the sense that the genomic sequences are compared to a random sequence sharing only the same AT content. Low *D_KL _*means low relative entropy and high *D_KL _*means high relative entropy [18]. Since the DNA sequence from the biological entity is compared to a random, 0^th ^order Markov based sequence (sharing only total AT content), a lower *D_KL _*reflects a greater independence between nucleotides in the corresponding tetranucleotides, and hence that less information is carried by the DNA sequence. Conversely, higher *D_KL _*is taken to mean that more information is carried by the DNA sequence since the adjacent nucleotides in the corresponding tetranucleotides are more dependent on each other.

We sought to use methods from information theory to examine information capacity (relative entropy) in chromosomes, plasmids, phages and GIs. We investigated possible influences affecting relative entropy in the different types of DNA sequences and how relative entropy varies along bacterial chromosomes, focusing particularly on the AT rich *Bacillus cereus*, the medium AT:GC *Escherichia coli *and the GC rich *Mycobacterium tuberculosis*. We also examined the relative entropy of highly conserved genes in two closely related species (*M.tuberculosis *and *M.leprae*) of which one has presumably undergone considerable genome reduction [19,20].

## Results

### A note on the calculation of D_KL_

The relative entropy of a DNA sequence, which we refer to as *D_KL_*, is measured as the divergence between observed tetranucleotide frequencies from approximated tetranucleotide frequencies using a zero order Markov model. The zero order Markov model assumes that every base in the sequence is occurring with a probability independent of all other neighboring bases. It is reasonable to assume that in regions of high mutation activity this is a good description [11]. We compare the computed tetranucleotide frequencies from the zero order Markov model to counted tetranucleotide frequencies from each DNA sequence. So the information capacity in a DNA sequence is positively associated with the magnitude of the divergence from the approximated sequence. Hence, the higher the divergence between observed and expected (approximated) tetranucleotide frequencies the more information potential in the DNA sequence, and vice versa.

### D_KL _differences between chromosomes, GIs, phages and plasmids

**Figure 1:**
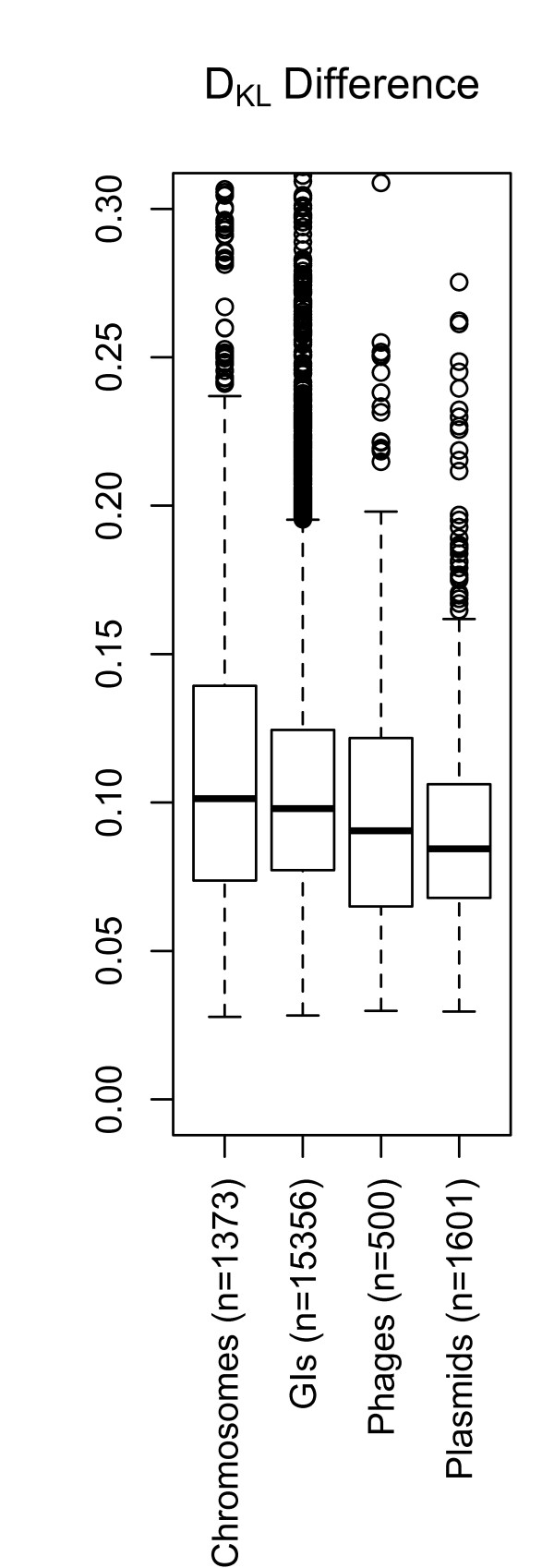
**Boxplot of *D_KL_***. The box and whisker plot shows *D_KL _*differences between chromosomes, GIs, phages and plasmids. The boxes contain 50% of the values surrounding the median (thick horizontal line), while the lower and upper whiskers represent approximately the 25% lowest and highest *D_KL _*values, respectively. The open circles above the upper whiskers are considered as outlying values. Although differences were small between the types of DNA sequence families, chromosomes were found to be, on average, the most biased (highest information potential) while plasmids the least.

We examined whether information capacity varied between chromosomes and two potential 'vectors': *i.e*. phages and plasmids, as well as GIs. Figure 1 shows that the *D_KL _*was slightly lower amongst GIs than chromosomes (*p~0.004*, see the Methods section for more details on the statistical methods). Phages were in turn found to have a lower *D_KL _*than GIs (*p < 0.001*), and plasmids had slightly lower *D_KL _*than phages (*p~0.004*). Hence, the largest difference in *D_KL _*(the most divergent tetranucleotide frequencies compared to a random sequence) was between chromosomes and plasmids (*p < 0.001*). In other words, chromosomes were, on average, the most biased DNA sequences while the plasmids had the most random (least biased) DNA composition.

### Relative entropy vs AT content

**Figure 2:**
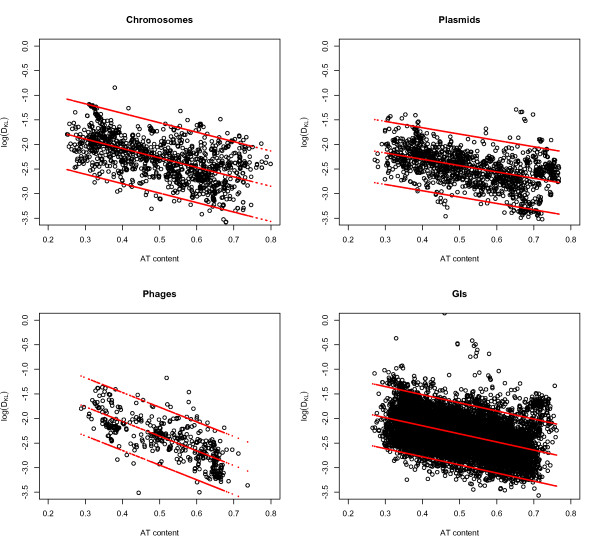
***D_KL_*vs AT content**. Log-transformed *D_KL _*(vertical axis) is plotted against AT content (horizontal axis) with accompanying regression lines and 99% prediction intervals for chromosomes, plasmids, phages, and GIs. A clear correlation can be observed for all DNA sequences between (log-transformed) *D_KL _*and AT content meaning that randomness in DNA sequences increases with AT content. The highest correlation was observed between relative entropy in phages and AT content.

An association between information capacity and AT content has been found for chromosomes in previous studies using slightly different methods than those described here (see Methods section) [10,11]. Since there was a statistical significant difference in relative entropy between vectors (plasmids and phages) and chromosomes we explored whether similar associations could be found between the vectors and AT content. Figure 2 shows that relative entropy, *D_KL_*, in chromosomes, plasmids, phages and GIs is negatively correlated with AT content: *D_KL _*tends to decrease with increasing AT content. Regression analyses with *D_KL _*as the response and AT content as the predictor gave *R^2 ^= 0.33 *for chromosomes, *R^2 ^= 0.21 *for plasmids, *R^2 ^= 0.56 *for phages, and *R^2 ^= 0.22 *for GIs. A likelihood ratio test between ANOVA models with size plus AT content versus AT content alone did not improve the correlation. All statistical results mentioned were significant, *p < 0.001*.

### Relative entropy comparisons of shared genes between *M. tuberculosis* and *M. leprae*

**Figure 3:**
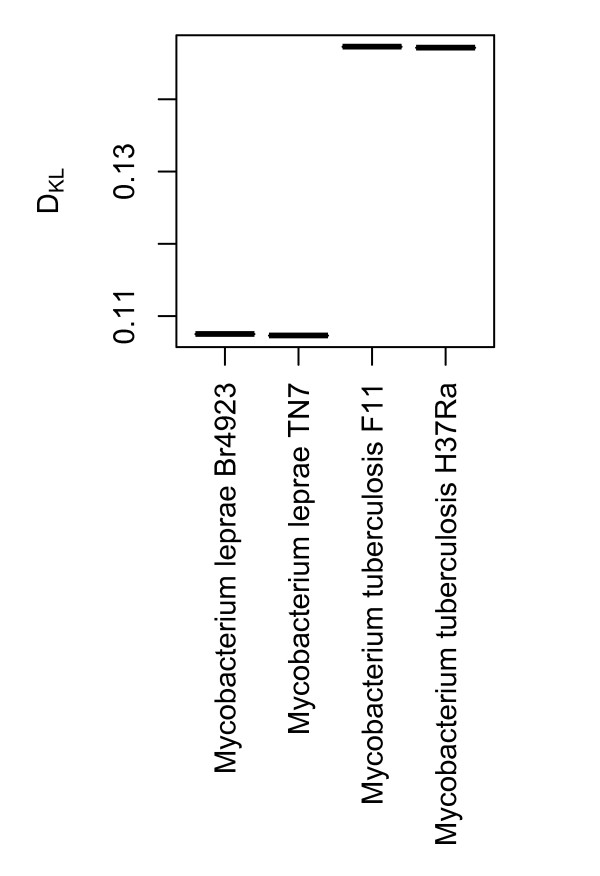
***D_KL_*differences in *M. leprae *and *M. tuberculosis***. The figure show *D_KL _*(vertical axis) for shared highly conserved genes of *M. leprae *and *M. tuberculosis *(horizontal axis).

It has been shown that the genomes of intracellular microbes have a tendency to reduce in size due in part to more mutations and eventual loss of DNA repair genes [21,22]. We examined whether these changes are reflected in relative entropy of the genomes of *M. tuberculosis*, a facultative intracellular pathogen, and *M. leprae*, an obligate intracellular pathogen considered to be in a transitional state between free living and intracellular lifestyles [19,20]. *M. leprae *has a smaller genome than *M. tuberculosis *(3.3 mb vs. 4.4 mb) and it is more AT rich (42.3% vs 24.4%). Figure 3 shows that *D_KL _*taken from highly conserved coding regions was also lower in *M. leprae *than for *M. tuberculosis*, implying that *M. leprae *has a more random base composition, possibly due to an increased number of accumulated mutations. The fact that relative entropy was taken from shared functional genes between the two organisms supports the existing model of genome decay in intracellular microbes [21] resulting in increased randomness amongst the protein coding regions.

### Phylogenetic influence on relative entropy

**Figure 4:**
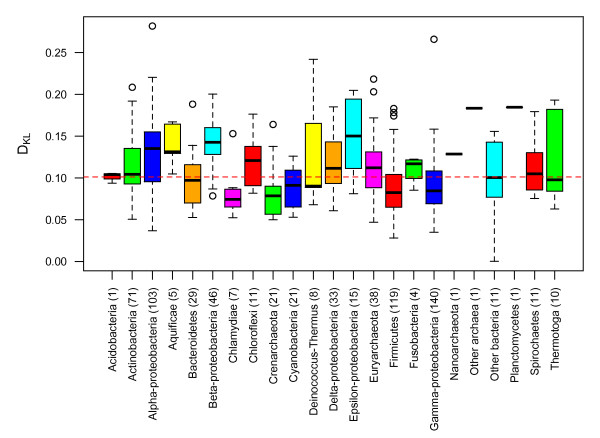
***D_KL _*vs Phyla**. The figure shows a boxplot of *D_KL _*(vertical axis) plotted against phylogenetic groups (horizontal axis, number of genomes from each phylogenetic group in parenthesis). The dashed horizontal red line is the average *D_KL _*for all groups.

Using comparable methods to *D_KL_*, Reva and Tümmler argued that DNA sequence bias appears to be a taxon-specific phenomenon within bacteria [10]. To assess whether *D_KL _*was influenced by taxonomy (Figure 4) we picked out one strain from each species to decrease bias from multiple strains, reducing the dataset to 709 chromosomes. We found that phylogenetic relationship did significantly influence *D_KL_*, but only slightly (*R^2 ^= 0.21*) and comparable to that of GC content (*R^2 ^= 0.22*). The phyla and %GC factors did, however, not interact and a model including both GC content and phyla as predictors explained approximately 40% (*R^2 ^= 0.4*) of the variance observed. All results were statistically significant with *p < 0.001*. No significant difference (*p~0.87*, Welch two-sample T test) in relative entropy was found between archaea and bacteria.

### D_KL _changes within genomes

**Figure 5:**
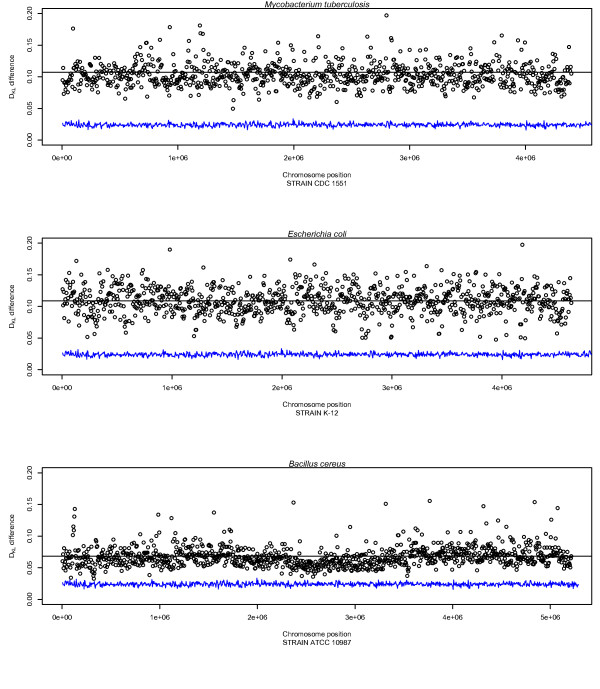
**Profiles of *D_KL _*differences within *M. tuberculosis, E. coli *and *B. cereus***. Profiles made from the *D_KL _*values of non-overlapping sliding windows in *M. tuberculosis, E. coli *and *B. cereus*. It can be seen that *D_KL _*values within the chromosomes are remarkably stable. *B. cereus *has noticeably lower *D_KL _*values than the other genomes indicating that the chromosome has a comparably more random base composition. The *D_KL _*values of a 50% GC content random genome are also included for comparison. For all chromosomes, the black horizontal line represents mean *D_KL_*.

To assess how relative entropy varied within bacterial chromosomes we examined the chromosomes of GC-rich *Mycobacterium tuberculosis *(65% GC), *Escherichia coli *K-12 with approximately 50% AT/GC, and AT rich *Bacillus cereus *(65% AT) using a sliding window of 5 kbp with *D_KL _*from each window compared to *D_KL _*for the whole chromosome. The aim was to examine whether *D_KL _*could be regarded as a stable measure within bacterial chromosomes, similar to the genome signature [23]. Figure 5 shows how *D_KL _*changed within the three species compared to a randomly constructed 50% GC chromosome of equivalent size to *E.coli *(5 Mbp). Notice that although *D_KL _*varied within the chromosomes the level of variance was stable, indicating that average *D_KL _*is a robust property for the whole DNA sequence.

**Figure 6:**
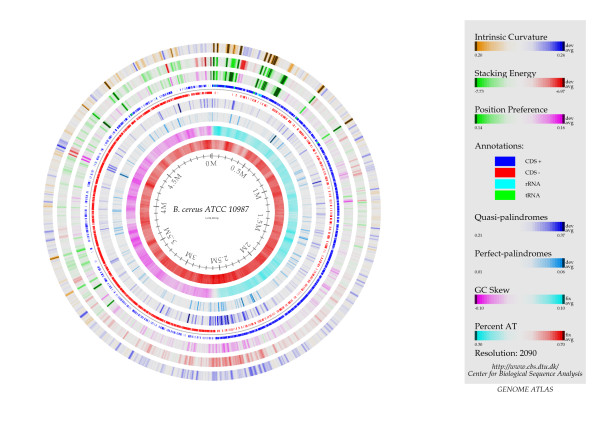
**Blast atlas of *B. cereus *ATCC 10987**. Blast atlas for *B. cereus *ATCC 10987 depicting several structural and sequential features. Of special interest is the region approximately between 2 mb and 3 mb, which has a substantial higher occurrence of both quasi- and perfect palindromes, increased stacking energy and intrinsic curvature, as well as higher position preference than the rest of the chromosome.

In addition, Figure 5 shows that although *M. tuberculosis *and *E. coli *had similar *D_KL _*measures throughout the chromosome, the *B. cereus *chromosome exhibited considerably lower *D_KL_*. This was especially pronounced in the middle of the chromosome. The accompanying BLAST atlas (Figure 6) [24] shows that the DNA molecule in this area was more AT rich, had more pronounced intrinsic curvature, increased stacking energy (making the double stranded DNA string easier to melt), higher position preference, and a higher occurrence of quasi- and perfect palindromes.

### Size vs AT content

**Figure 7:**
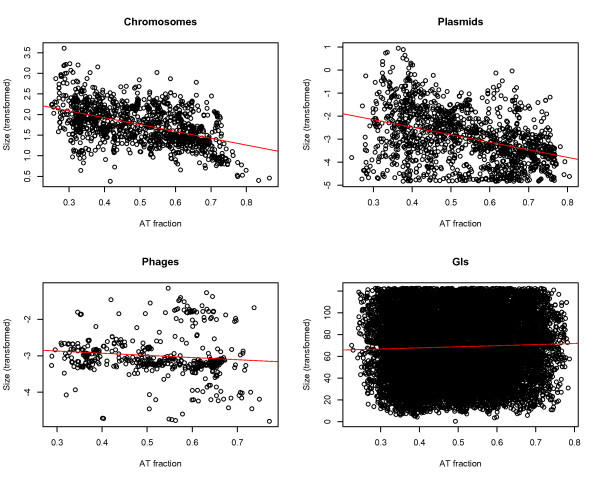
**Size vs AT content**. The Figure depicts DNA sequence size from chromosomes, phages, plasmids and GIs (vertical axis) plotted against their corresponding AT contents (horizontal axis). Associations between DNA sequence size and AT content can be observed for chromosomes and plasmids.

Although it has been demonstrated that AT content and chromosome sizes are inversely correlated in prokaryotes, we carried out additional tests for plasmids, phages, GIs as well as chromosomes. From Figure 7 it can be seen, as expected, that we found an association between chromosome size and AT content *R^2^~0.22, p < 0.001*. In addition, we found a significant association between plasmid size and AT content, albeit low (*R^2^~0.16*), which could be due to the increased variance. With an *R^2^~0.01 *or less, the size of both phages and GIs were not associated with AT content. All results were statistically significant (*p < 0.001*).

### Size vs. relative entropy

Since the correlation between DNA sequence size and GC content is well established [25,26] we examined whether *D_KL _*was affected by DNA sequence size. We performed regression analyses with *D_KL _*of chromosomes, GIs, phages and plasmids as the response and the corresponding sequence size as the predictor variable, measuring, in effect, the correlation between *D_KL _*and sequence size. In all instances *R^2 ^*(the coefficient of determination) was found to be lower than 0.05, meaning that less than 5% (*p < 0.001*) of the variance observed in the data was explained by the regression models. A regression analysis with GC content as outcome indicated that variance explained increased additively as DNA sequence size (21% and 15% (*p < 0.001*) for bacterial chromosomes and plasmids, respectively) and *D_KL _*(48% and 29% (*p < 0.001*) chromosomes and plasmids, respectively) was added to the model. Hence, AT content has an independent effect on DNA sequence size and relative entropy in bacterial chromosomes and plasmids, while *D_KL _*was not affected by DNA sequence size regardless of DNA sequence type examined. It should be noted that for the combined regression model including both *D_KL _*and DNA sequence size the %-variance explained metrics (i.e. *R^2^*) were slightly different from the individual models discussed in the above sections due to the different types of transformations used (see Materials section for further details).

## Discussion

### Relative entropy in chromosomes, plasmids, phages and GIs

Chromosomes were, on average, the most biased sequences (*i.e*. least similar to a random sequence) and therefore presumably the most subjected to selective pressures of the sequences examined here. In terms of *D_KL _*there was a small, but significant difference between GIs and chromosomes. This difference is expected since GIs are found within chromosomes and have ameliorated over time, which, in base compositional terms, tend towards that of the host chromosome [27]. Hence, a number of studies indicate that GIs consist of horizontally acquired mobile genetic fragments [22,28], but our data does not identify what type of vector has brought these GIs to their respective chromosomes.

The reduced *D_KL _*of phages compared to plasmids was small but statistically significant. In contrast to phages, plasmids exist independently of the host chromosome and are generally non-lethal [29]. When the phenotypic features of the plasmid are not required for bacterial survival, the plasmid will exist only in a small minority of the total microbial population [30]. In this way the forces of selective pressure are reduced compared to the host chromosome. Phages also exist independently of bacterial chromosomes but rely on the bacterial machinery for replication [29,30]. However, those phages that are lytic will be under greater selective pressure than plasmids. What particular features of phages that result in the reduced information content remains to be clarified.

It should be noted that the comparisons were between all deposited DNA sequences, which means that the results reflect the distributions of chromosomes, GIs, phages and plasmids that initially have been originally selected and sequenced for a purpose. The effect of this bias is not clear.

### Association between D_KL _and AT content

Figure 2 shows that decreased relative entropy *(D_KL _*) is associated with increasing AT content. An example of this was demonstrated in Figure 3, where the more AT rich *M. leprae *was found to have lower *D_KL _*in genes that are also shared with the more GC rich *M. tuberculosis*.

Although the coefficient of determination, *R^2^*, varied between GIs, phages, plasmids and chromosomes, Figure 2 shows that the trend remained for all DNA sequences examined. Phages obtained a surprisingly high coefficient of determination, *R^2 ^= 0.56*, implying that relative entropy was more linked to changes in AT content in these organisms.

### D_KL _variation within chromosomes

The *D_KL _*profile of the *B. cereus *chromosome may imply that areas of low relative entropy (low *D_KL_*) might be indicators of genetic regions especially prone to rearrangement. This propensity for re-arrangements may be due to the increased stacking energy, position preference and amount of quasi-palindromes observed in the region, all of which are determinants of genomic re-arrangement. The relatively high occurrence of both palindromes and quasi-palindromes in the region of *B. cereus *with low relative entropy may indicate that the mechanisms leading to quasi palindrome correction have not been operating properly in these regions as compared to the chromosome in general [31] possibly resulting also in a higher number of accumulated mutations [17]. A similar region has been found for all sequenced members of the *B. cereus*-group, which implies that the genetic region has been selected and kept possibly due to some unknown advantage. As can be seen from Figure 6, the region is predominantly gene coding. Since the genomes of the *B. cereus *group are relatively large compared with the distantly related *B. subtilis *it can be speculated that the region is an acquired phage or plasmid.

### Connections between DNA sequence and structure

Although relative entropy has some mathematical associations with thermodynamics the two concepts are, in general, independent of each other [18]. However, it is known that greater energy is required to melt GC rich sequences than AT rich sequences [32]. Considering our results found a negative correlation between *D_KL _*and AT content it is possible that DNA structure energetics and DNA sequence relative entropy may be connected and provides a link between DNA structure and sequence. This is supported by the findings shown in Figure 6 where a genetic region of low relative entropy was found to have more intrinsic DNA structural curvature, increased stacking energies and higher position preference. Hence, our findings may point to possible DNA structural differences between bacterial chromosomes, plasmids and phages that could have implications for how these biological entities are integrated into their hosts.

### Phylogenetic influences on relative entropy

Our measure of relative entropy revealed that approximately 21% of the variation in *D_KL _*could be explained by a close phylogenetic relationship. This value compares well with the 22% in variation that is explained by GC content. Thus, *D_KL _*appears to be as much influenced by phyla as GC content is, while almost 80% is accounted for by other factors. Using a method that is strongly associated with relative entropy (OUV, oligonucleotide usage variance), 55% of the variance could be explained by environment, phyla and AT content [17]. If non-coding regions were excluded 67% of the variance could be explained using environment, phylum and AT content. The above mentioned study also discusses possible influences between environmental factors and possible implications of high and low OUV for a number of microbes that is relevant to the present exposition. The difference between OUV and relative entropy is explained in the Methods section.

### Relation between relative entropy and DNA sequence size

Although a possible link between plasmid size and ecology has been reported [29], and a correlation between microbial chromosome size and GC content has been established previously, to the best of our knowledge no such correlation has been reported between plasmid size and GC content. It can also be seen from Figure 7 that plasmid sizes vary considerably more with respect to AT content than chromosomes, which could indicate that the DNA sequences of plasmids are less stable and more prone to genetic exchange than the DNA sequences of chromosomes.

### Lack of correlation between relative entropy and DNA sequence size

Although a correlation between DNA sequence size and *D_KL _*in bacterial chromosomes and plasmids could be expected due to the correlation found between these factors and genomic AT content, no such correlation was found. This may imply that the relation between genomic AT content and DNA sequence size is independent of the relation between genomic AT content and relative entropy. In other words, genomic AT content may be differently related to DNA sequence size than to relative entropy in bacterial chromosomes and plasmids (no correlation was found between AT content and DNA sequence size in GIs and phages). This claim was further strengthen by a linear regression analysis, which indicated that the variance explained increased additively with DNA sequence size and relative entropy added as predictors. Hence, our models indicate that the mechanisms connecting AT with DNA sequence size are unrelated and different to the mechanisms linking AT content with relative entropy.

### Connections to other studies

By using BLAST and graph/network analyses it has been found that the different groups, *i.e*. chromosomes, plasmids and phages, share, in the majority of cases, DNA amongst themselves. In other words, chromosomes share DNA with chromosomes, plasmids share DNA with plasmids and phages share DNA with phages [5]. Variation among bacterial chromosomes however is predominantly mediated by genetic exchange from plasmids and only transiently so by phages [5]. Our results indicated that plasmids, on average, had significantly lower *D_KL _*than any of the other types of DNA sequences. This could mean that plasmids are more tolerant to genetic alterations something that may be crucial to maximize host range [33]. A previous study has reported a correlation between plasmid-host similarity and GC content, *i.e*. the more similar the plasmids-hosts were in terms of genomic signatures, the more GC rich they tended to be [9]. Phages have been found to have a narrow host range, in fact even more so than plasmids [5] in spite of their larger numbers (estimations go as high as 5-10 phages for each bacterium on earth [34-36]), which may indicate that they have been subjected to increased selective pressures resulting, in turn, in significantly higher *D_KL _*than for plasmids. Due to the possible link between relative entropy and DNA sequence mutations it can be speculated whether phages are more vulnerable to genetic rearrangements than plasmids, resulting in higher *D_KL_*, on average in phages.

## Conclusions

In conclusion, we find that GIs and chromosomes have similar relative entropy (*D_KL_*), which may be due to amelioration of the foreign DNA towards the base composition of the host chromosome. Both plasmids and phages had significantly lower relative entropy than GIs and chromosomes. Plasmids had the lowest *D_KL _*of all types of DNA sequences examined, meaning that plasmids contained, on average, the most mutated DNA sequences. Relative entropy decreased in all types of DNA sequences in concordance with increasing AT content, possibly implying that the number of accumulated mutations appear to increase with AT content regardless of the (prokaryotic) biological entity. This was also demonstrated on a shared set of highly conserved genes from *M. tuberculosis *and *M. leprae*, of which the latter, known to have undergone considerable genome reduction, was found to have significantly lower relative entropy (*i.e*. more random DNA sequences possibly due to mutation) in the protein coding genes. AT content and *D_KL _*association was especially pronounced for phages, which may reflect an evolutionary strategy that associates the number of accumulated mutations with AT content to a substantially larger extent in phages than bacteria.

## Methods

Chromosomes, plasmids and phages were downloaded from the NCBI website http://www.ncbi.nlm.nih.gov/genome/, while the GIs were downloaded from the Islandviewer website http://www.pathogenomics.sfu.ca/islandviewer/query.php. Only DNA sequences larger than 10 kb were considered due to limitations of the method. Single copy orthologs were assigned by OrthoMCL [37] for the genomes of *Mycobacterium tuberculosis *F11 (CP000717.1), *M. tuberculosis *H37Ra (AL123456.2)*, M. leprae *Br4923 (FM211192.1) and *M. leprae *TN7 (AL450380.1). Statistical analyses were carried out with R http://www.r-project.org/, which was also used to create all figures except the BLAST atlas (Figure 6). The BLAST atlas was made using CBS in-house software [24,38].

The Kullback-Leibler divergence (*D_KL_*, also referred to as the relative entropy) is a measure of difference between two discrete probability mass functions [18]. Let **s **be a DNA sequence, and **z**_1_,...,**z**_256 _be all possible tetramers of the DNA alphabet (4^4 ^= 256). The observed frequencies of tetranucleotides from DNA sequence **s **is written as *O*(**z**_i_|**s**). The expected frequencies of tetranucleotides from DNA sequence **s **found using a zero order Markov model is written as *E*(**z**_i_|**s**). The KL divergence for the sequence **s **is given as:

$$D_{KL}\left(s\right)=\overset{256}{\underset{i=1}{\sum }}O\left(z_{i}|s\right)\log\left(\frac{O\left(z_{i}|s\right)}{E\left(z_{i}|s\right)}\right)$$

A lower *D_KL _*is interpreted as lesser information potential is carried by the DNA sequence **s **due to lesser dependence between the nucleotides in the corresponding tetranucleotides. Conversely, a higher *D_KL _*is taken to mean that higher information potential is carried by the DNA sequence (higher relative entropy), since the nucleotides in the corresponding tetranucleotides are more dependent on each other. The OUV measure [17] described in the Discussion section and compared to relative entropy is calculated as follows (*O, E*, **z**_i _and **s **are the same as above):

$$D_{OUV}\left(s\right)=\frac{1}{256}\overset{256}{\underset{i=1}{\sum }}\frac{O\left(z_{\text{i}}|s\right)}{E\left(z_{\text{i}}|s\right)}$$

Although the OUV measure is similar to relative entropy, we use the latter here due to the larger theoretical framework and tools available from information theory [12,18].

Comparisons between *D_KL _*and factors such as phyla, AT content, DNA sequence size, etc. were carried out using linear regression with transformations applied to correct for non-normality where needed.

*D_KL _*was computed for each DNA sequence (chromosome, plasmid, phage and GI) and compared to AT content, size and phyla using linear regression:

Y=a+bX+ε

For comparisons between chromosome, plasmid, GI and phage size (*Y = Y_size_*) versus *D_KL _*(*X_KL_*) no transformation was used.

To examine the relationship between *D_KL_*, DNA sequence size and AT content for bacterial chromosomes and plasmids, a linear regression model was used without transformations on the response:

$$Y_{AT}=a+bX_{KL}+cX_{Size}+d{\left(X_{Size}\right)}^{2}+\varepsilon$$

Linear regression between *D_KL _*as outcome (*Y = Y_KL_*) and AT content as response (*X = X_AT_*) was log-transformed:

$$LogY_{KL}=a+bX_{AT}+\varepsilon$$

Several transformations were used to assess associations between chromosome, plasmid, phage and GI size (*Y_Size_*) vs AT content (*X_AT_*) using the following regression equation:

$$Y_{Size}=a+bX_{AT}+\varepsilon$$

A square root transform was used when the response was sequence sizes for chromosomes; log transformations for both phage and plasmid sizes; and (1/ *Y_Size_*) transform for GI sizes as outcome.

Comparison of *D_KL _*between chromosomes, plasmids, phages and GI, as seen in Figure 1, were carried out using the non-parametric Wilcoxon (Mann-Whitney) test due to skewed (but similar) distributions.

All statistical results presented as results were found to be statistically significant with *p < 0.001*, if not otherwise stated in the text.

All *D_KL _*measurements of DNA sequences were carried out using in-house software. The profiles measuring *D_KL _*changes within bacterial chromosomes as seen in Figure 5 were performed using non-overlapping sliding windows of 5 kbp compared to average chromosomal *D_KL_*.

## Authors' contributions

JB, LS and ABK carried out statistical analyses. JB, MWJvP, SPH, and DU contributed to data analyses and discussion. All authors participated in the writing of the manuscript. The study was initiated by JB. All authors have read and approved the final manuscript.
